# Do Neuroendocrine Peptides and Their Receptors Qualify as Novel Therapeutic Targets in Osteoarthritis?

**DOI:** 10.3390/ijms19020367

**Published:** 2018-01-26

**Authors:** Susanne Grässel, Dominique Muschter

**Affiliations:** Department of Orthopedic Surgery, Exp. Orthopedics, ZMB/Biopark 1, University of Regensburg, 93053 Regensburg, Germany; dominique.muschter@ukr.de

**Keywords:** proopiomelanocortin, alpha-MSH, VIP, NPY, osteoarthritis, neuroendocrine, PACAP

## Abstract

Joint tissues like synovium, articular cartilage, meniscus and subchondral bone, are targets for neuropeptides. Resident cells of these tissues express receptors for various neuroendocrine-derived peptides including proopiomelanocortin (POMC)-derived peptides, i.e., α-melanocyte-stimulating hormone (α-MSH), adrenocorticotropin (ACTH) and β-endorphin (β-ED), and sympathetic neuropeptides like vasoactive intestinal peptide (VIP) and neuropeptide y (NPY). Melanocortins attained particular attention due to their immunomodulatory and anti-inflammatory effects in several tissues and organs. In particular, α-MSH, ACTH and specific melanocortin-receptor (MCR) agonists appear to have promising anti-inflammatory actions demonstrated in animal models of experimentally induced arthritis and osteoarthritis (OA). Sympathetic neuropeptides have obtained increasing attention as they have crucial trophic effects that are critical for joint tissue and bone homeostasis. VIP and NPY are implicated in direct and indirect activation of several anabolic signaling pathways in bone and synovial cells. Additionally, pituitary adenylate cyclase-activating polypeptide (PACAP) proved to be chondroprotective and, thus, might be a novel target in OA. Taken together, it appears more and more likely that the anabolic effects of these neuroendocrine peptides or their respective receptor agonists/antagonists may be exploited for the treatment of patients with inflammatory and degenerative joint diseases in the future.

## 1. Introduction

Clinical symptoms of osteoarthritis (OA) appear in more than 10% of the world population and affect almost everyone over the age of 65. As a consequence of the increasing longevity and obesity within the European Community, the economic and social burden caused by OA is growing rapidly and substantially influences the life quality of the affected individuals causing enormous costs to the health care system for diagnosis, treatment, sick leave, rehabilitation, and early retirement [1]. A recent survey in 15 European countries revealed that, on average, 19% of the population suffer from chronic pain, most frequently caused by disorders of the musculoskeletal system, specifically OA, herniated and/or deteriorating discs, traumatic injury and rheumatoid arthritis [2]. This study also documented, that a majority of patients with pain experience sleep disturbances and a reduced ability to exercise, lift, and walk. Moreover, chronic pain may prevent attending social activities, to work in and outside the house, to maintain an independent life style, to have a normal sexual life, etc. The burden of OA not only includes physical problems, it also has detrimental psychological effects. Psychological distress is more frequently experienced by patients with OA compared to patients with other chronic diseases such as diabetes [3].

Current pharmacological strategies either seek to relieve pain and increase mobility (symptom modifying drugs) or aim to affect the disease (DMOAD, disease modifying osteoarthritis drugs). Pain is treated with non-steroidal anti-inflammatory drugs, cyclo-oxygenase (COX)-2 inhibitors, opioids, corticosteroids, viscosupplementation and symptomatic slow-acting drugs [4]. To date, none of the current DMOAD-based approaches was able to stop disease progression, nor regenerate damaged cartilage in the long term [5]. The OARSI guidelines recommend glucosamine- and chondroitin sulfates and diacerein as DMOADs, and the National Institute for Health and Clinical Excellence will recommend glucosamine sulfate in the next update of their guidelines. Especially, glucosamine derivatives are promising DMOADS and show a highly protective capacity in OA animal models after intra-articular injection [6]. Exploration of improved outcome measures and identification of subgroups of patients benefiting from different DMOADs are the most important areas of research for the coming years [7,8]. 

Often this treatment is insufficient and, finally, after many years of pain, most patients have to undergo surgical joint replacement therapy, e.g., total knee arthroplasty. Thus, there is an ultimate need for the development of new, non-invasive or minimal-invasive treatments that could slow down and/or stop OA progression and substitute joint replacement for end-stage OA-patients. These may include therapeutic options with neurohormones like proopiomelanocortin (POMC)-derived peptides and neuropeptides of the sympathetic and parasympathetic nervous system.

## 2. Biology of POMC-Derived Peptides and Their Receptors 

### 2.1. POMC Gene Expression and Processing

POMC is a multifunctional precursor protein for several biologically active hormones which include adrenocorticotropin (ACTH), the melanocyte-stimulating hormones (α-, β- and γ-MSH) and the endogenous opioid β-endorphin (β-ED). The human POMC gene is composed of three exons and two large introns. Exon 1 is untranslated while parts of exon 2 code for the signal sequence. Exon 3 codes for the bioactive POMC-derived peptides which are generated after proteolytic processing of POMC [9]. Only the full length POMC transcript (1200 bp) is functional, smaller transcripts are neither processed nor secreted. Glucocorticoids acting via glucocorticoid-responsive elements repress POMC transcription whereas in peripheral cell types pro-inflammatory stressors including ultraviolet light irradiation, pro-inflammatory cytokines such as interleukin (IL)-1 and tumor necrosis factor (TNF)-α and the tumor suppressor gene product p53 turn on POMC expression [10]. Epigenetic mechanisms involving hypermethylation of a CpG-rich island upstream of exon 1 in the 5′ POMC promoter region control POMC gene expression [11], however, in many peripheral tissues the POMC promoter is permanently methylated. After translation, the ~30 kDa POMC precursor protein is further processed by prohormone convertases (PCs) [12]. PC1 cleaves POMC into ACTH and β-lipotropic hormone whereas PC2 further processes ACTH and β-lipotropic hormone to the various MSHs (α-MSH, γ-MSH and β-MSH) as well as β-endorphin (β-ED). Although POMC is considered a precursor protein for the above listed biologically active peptide hormones it should be noted that full-length POMC also possesses weak melanotropic activity in vitro [13]. 

### 2.2. POMC-Derived Peptide Receptors

The melanocortin peptides ACTH and α-, β- and γ-MSH bind with high affinity to melanocortin receptors (MCRs) which belong to the superfamily of G-protein coupled receptors (GPCRs) with seven transmembrane domains [14]. Five MCR subtypes, MC1R to MC5R, have been cloned which have a sequence homology of 39–61% to one another at the amino acid level. The MCR subtypes bind melanocortins with different affinities. MC1R binds both, ACTH and α-MSH, with almost equal affinity while MC2R is selective for ACTH. However, ACTH also binds to MC3R, MC4R and MC5R, although γ-MSH is the preferred ligand for MC3R and α-MSH the preferred ligand for the MC5R. Signal transduction is mediated via increase in cyclic adenosine monophosphate (cAMP) and also Ca^2+^ mobilization from intracellular stores [12]. For in depth description of melanocortin receptors, their regulation of activity and signal transduction pathways see the recent review from Montero-Melendez [15]. In the presence of pro-inflammatory co-stimuli such as IL-1 or TNF-α, melanocortin peptides suppress signal transduction pathways linked to inflammation and immune responses. This has been demonstrated for various MC1R-expressing cell types in which α-MSH can significantly attenuate activation of the redox-sensitive nuclear factor-κB (NF-κB) [16].

β-ED has high affinity for both the µ-opioid receptor (MOR) and δ-opioid receptor (DOR); both are G-protein coupled receptors. In analogy to MCRs, MORs and DORs have been detected in an increasing number of extra-neural tissues and non-neuronal cells indicating functions of β-ED far beyond anti-nociception [17]. The signaling events elicited by β-ED are complex and mediated via more different intracellular pathways compared to MCR-mediated responses and are not the focus of this review.

## 3. Biology of Sympathetic Neuropeptides and Their Receptors 

The classical neurotransmitters of sympathetic and parasympathetic nerves are noradrenaline (NE) and acetylcholine (ACh), respectively. Both types of nerve fibers can be accompanied by peptidergic transmitters and some nerve fiber types predominantly express neuropeptides as their main transmitter. Catecholaminergic sympathetic neurons often co-express neuropeptide Y (NPY) [18,19], whereas ACh co-localizes with vasoactive intestinal peptide (VIP) [20,21]. Alongside, peptides like pituitary adenylate cyclase-activating peptide (PACAP) and enkephalin and somatostatin have been described in the sympathetic nervous system [22,23]. In this review, we will focus on the peptidergic co-transmitters of peripheral sympathetic and parasympathetic nerves, especially in the musculoskeletal system.

### 3.1. Neuropeptide Y

NPY is a 36-amino-acid peptide first isolated and sequenced from porcine brain extracts in 1982 by Tatemoto et al. [24]. The highly homologous neuropeptide Y family of biologically active peptides also includes peptide YY (PYY) and the pancreatic polypeptide (PP) sharing a characteristic of high numbers of tyrosine residues in their sequence [25]. Beside its manifold functions in the central nervous system, peripheral actions involve modulation of inflammatory or neuropathic pain [26], regulation of the gut-brain axis [27] and the cardiovascular system [28,29] as well as angiogenesis [30]. NPY is also involved in the metabolic syndrome [31], influences proliferation and migration of cells of various origins [32,33,34] and possesses important immune-modulatory functions [35].

Six different receptor subtypes were identified and named Y1–5 and y6. Existence of Y3 has been questioned and a functional receptor subtype y6 has so far only been detected in mice and rabbits, whereas it is absent in rats and inactive in humans and pigs due to frame-shift mutations [36]. A very elaborate review on NPY receptor signaling was published by Michel et al. in Pharmacological Reviews in 1998 [37]. All of the Y receptors belong to the pertussis toxin-sensitive G_i_ or G_0_ types of the GPCR family mediating adenylate cyclase (AC) inhibition [38,39]. Pertussis-toxin insensitive receptor activity of NPY was observed in a small number of studies [40]. Furthermore, NPY receptor activation was demonstrated to increase intracellular calcium concentration and inositol 1,4,5-triphosphate (IP_3_) production [41,42], inhibit voltage-dependent calcium influx [43] and block nicotinic cholinergic currents [44].

### 3.2. Vasoactive Intestinal Peptide and Pituitary Adenylate Cyclase-Activating Peptide

VIP and PACAP belong to the glucagon/secretin superfamily of peptide hormones consisting of nine peptidergic members. PACAP and VIP show high sequence homology and hence share similar biological activity/effects. PACAP38 is the most ancient member of the family and has been characterized as a 38-amino-acid peptide from ovine hypothalamic extracts due to its ability to stimulate cAMP formation in anterior pituitary cells [45]. A shorter variant was isolated subsequently and termed PACAP27, corresponding to the 27 amino acids from the N-terminal region of PACAP38 [46]. PACAP27 shares 68% sequence homology with human VIP identifying it as a member of the secretin/glucagon family [47]. VIP has first been isolated from porcine small intestine by Said and Mutt in 1970 and has been characterized as a systemic vasodilator [48]. VIP is a 28-amino-acid linear peptide derived from a 170 amino acid prepro-peptide also giving rise to other biologically active peptides [49,50,51]. PACAP and VIP exert a huge range of biological functions that will exceed the scope of this review and, therefore, we refer the reader to respective general reviews in the literature [47,52,53,54]. The current evidence regarding PACAP/VIP-mediated effects in the musculoskeletal and immune systems will be elucidated more in detail in the following sections. VIP and PACAP exert their biological actions via common receptor types. The VIP/PACAP receptors (VPAC) 1 and 2 have similar affinities for both neuropeptides. PACAP additionally has a 1000-fold higher affinity for the PACAP receptor 1 (PAC1). VPAC 1 and 2 differ in their tissue distribution and are expressed differentially during developmental stages. VPAC and PACAP receptors signal via G_s_ protein-coupled receptors that activate AC and lead to cAMP accumulation and subsequent protein kinase A (PKA) activation. By coupling to G_q_ or G_i_, they might also activate phospholipase C (PLC) which in turn increases Ca^2+^ levels via IP_3_ and protein kinase C (PKC) activation via diacylglycerol (DAG) (reviewed by [55,56,57]). PAC1 receptor exists in different variations due to alternative splicing including hip- and hop-cassettes. These variations can either bind to AC and PKC, or to AC alone, resulting in different signal transduction options [58].

## 4. Joint Tissues as Target of the Neuroendocrine System 

### 4.1. Synovium 

The synovium includes the synovial membrane and the synovial fluid. The synovial membrane is a thin cellular layer that lines the joint cavity acting as a semipermeable filter to regulate the transfer of molecules in and out of the joint. It is a major source of the synovial fluid components, including nutrients, hormones and lubricant factors, such as lubricin and hyaluronic acid. Lubricant factors contribute to the unique low-friction properties of the articular surface [59]. Various synovial cell types exert specific functions within the osteoarticular system, e. g., extracellular matrix production, cytokine secretion, host defense and immunomodulation, to maintain the function of the joint under physiological conditions [60].

#### 4.1.1. POMC Peptides

In the recent past, POMC peptides were detected in the synovial fluid. A well-known study reported an increase of β-ED in synovial fluid of RA patients compared to OA patients [61]. In addition to β-ED, α-MSH was identified in the synovial fluid of patients with rheumatoid arthritis (RA), OA and juvenile chronic arthritis [62]. The levels of α-MSH were higher in patients with RA than in those with OA. Moreover, in patients with polyarticular/systemic-onset juvenile chronic arthritis the concentrations of α-MSH in the synovial fluid were higher than in those with pauciarticular disease. Recently, it was reported that synovial fluid α-MSH levels showed an independent and negative correlation with disease severity in patients with posttraumatic ankle osteoarthritis (PTAOA) according to Mankin scores, and degradation biomarkers CTX-II and AGG-1, as well as inflammation markers IL-6 and matrix metalloproteinase (MMP)-3 [63]. Recently, our group reported that melanocortin peptides like α-MSH act as endogenous response modifiers within the synovium. The POMC peptides previously detected in synovial fluid appear to derive either from the systemic circulation or from non-fibroblast cell types of the synovial membrane (e.g., macrophages or endothelial cells) since OA-synovial fibroblasts (OASF) do not synthesize POMC protein [64]. Importantly, various cell types within this tissue express receptors for POMC-derived peptides. Our group demonstrated that the MC1R is the only receptor for α-MSH and related peptides in OASFs [64]. We have also shown that α-MSH elicits biological effects in these cells suggesting an endogenous immunomodulatory role of melanocortins within the synovium.

#### 4.1.2. Sympathetic Neuropeptides

Synovial tissue is richly innervated and might serve as a source for various neurotransmitters and neuropeptides of sensory and sympathetic origin. Nerve fibers positive for the sympathetic marker enzyme tyrosine hydroxylase (TH) and NPY were detected in human synovium. In comparison to rheumatoid synovium, the expression in normal synovium was stronger [65]. While initial work postulated a loss of sympathetic innervation in the synovium of RA patients in relation to normal and OA synovium [66], Eitner et al. demonstrated the loss of sympathetic nerves in the inflamed OA synovium in contrast to synovial structures with little inflammation [67]. TH-positive nerves co-localized with NPY were found in the synovium of Sprague-Dawley rats adjacent to or within blood vessel walls and also in co-localization with IL-1, whereas VIP-immunoreactivity was seen in varicose nerve terminals and within vessel walls [68,69]. Synovial NPY-immunoreactivity increased slightly in Lewis rats with adjuvant-induced arthritis [70]. Equine synovium displays perivascular NPY in the subsynovium and vessel-associated expression in the fronds [71]. Nerve fibers positive for TH, NPY and VIP were also found in the normal guinea pig knee joint synovium [72]. NPY-immunoreactivity in the synovial tissue of various species confirms availability of this peptide in the joint cleft and might indicate that NPY could contribute to joint-associated diseases. In concordance with that assumption, synovial fluid concentrations of NPY show strong correlation with inflammation in human and animal studies in knee and temporomandibular joint (TMJ) arthritis [73,74,75]. NPY synovial fluid concentrations might therefore be a good biomarker candidate in OA. A study including 100 OA patients with varying grades of Watanabe pain scores and radiographic stages demonstrated that increasing synovial fluid concentrations of NPY positively correlated with pain scores. NPY concentrations in OA patients were significantly higher compared to controls and NPY synovial fluid concentrations of middle and advanced stage OA patients were higher compared to early-stage OA patients [76]. Furthermore, NPY expression increased in the dorsal root ganglion in monoiodacetate-induced OA [77].

**Figure 1:**
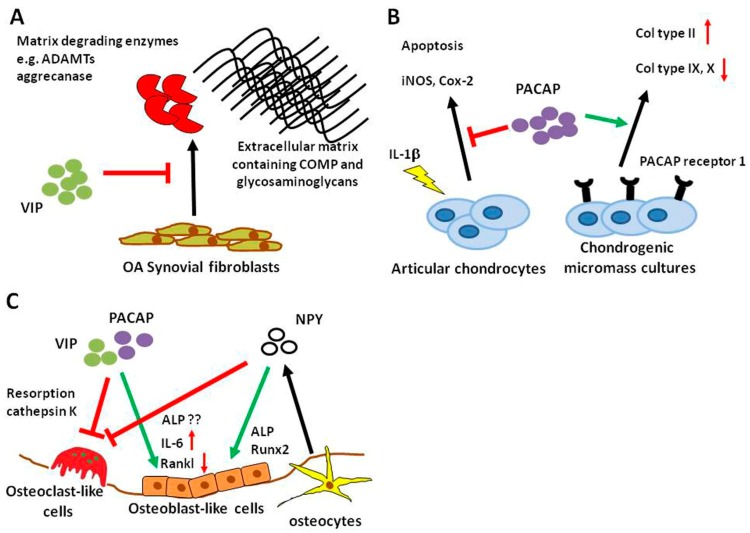
Effects of sympathetic neuropeptides on cells of the musculoskeletal system. (**A**) Vasoactive intestinal peptide (VIP) inhibits release of matrix-degrading enzymes a disintegrin and metalloproteinase with thrombospondin motifs (ADAMTs) and aggrecanase from osteoarthritis (OA) synovial fibroblasts and prevents degradation of cartilage oligomeric matrix protein (COMP) and glycosaminoglycans. (**B**) Chondrogenic micromass cultures express pituitary adenylate cyclase-activating peptide (PACAP) receptor 1. Stimulation with PACAP induced collagen type II production and inhibited hypertrophic markers collagen type IX and X. In chondrocytes derived from articular cartilage, PACAP reduced the interleukin (IL)-1β-induced induction of apoptosis and pro-inflammatory proteins inducible NO synthase (iNOS) and cyclooxygenase-2 (Cox-2). (**C**) VIP and PACAP inhibit osteoclast-mediated bone resorption and are able to induce IL-6 production in osteoblast-like cells. Inhibition of receptor activator of NF-ĸB ligand (Rankl) might support the inhibitory effect on osteoclastogenesis. Osteocytes can serve as a local source for neuropeptide Y (NPY) in bone, where it might inhibit osteoclastogenesis and enhance osteoblast formation and activity. ALP—alkaline phosphatase; red arrow—inhibition; green arrow—activation.

VIP-expressing nerves were detected in the cat synovial tissue, normal and arthritic mouse synovium and periost, as well as in rat synovium [68,78,79]. There have been several studies indicating that VIP has beneficial effects in synovial cell-derived joint morbidities. In OASF, VIP prevented ADAMTs (a disintegrin and metalloproteinase with thrombospondin motifs) production, inhibited aggrecanase activity and degradation of cartilage oligomeric matrix protein and glycosaminoglycan especially after stimulation with fibronectin fragments (Figure 1A) [80]. VIP levels were reduced in RA synovial fibroblasts (RASF) compared to OASF cells [81] and VIP stimulation inhibited the pro-inflammatory phenotype of RASF [82,83,84]. Synovial fluid concentrations of VIP were elevated in RA compared to controls and OA [85]. Research on the protective role of VIP on different joint tissues is predominantly restricted to studies in inflammatory arthritis models. The usefulness of VIP as a DMOAD deserves more intense investigations because available studies so far emphasize VIP as a potential inhibitor of joint degradation.

Concomitant to NPY and VIP, PACAP-containing nerve fibers were detected in the synovial membrane and joint capsule of the TMJ of the rat [86]. PACAP concentrations were reduced compared to controls in the synovial fluid and cartilage of rats with experimental OA [87]. The synovial tissue is a potent source for bioactive neuropeptides like NPY, VIP and PACAP that have the ability to influence joint cell metabolism in degenerative conditions like OA. An indicator for a putative role of the neuropeptides is the observed fluctuating expression during disease progression.

### 4.2. Cartilage 

Hyaline cartilage is a highly specialized connective tissue of mesenchymal origin. The only cell type within this tissue is the chondrocyte embedded in its self-contrived extracellular matrix (ECM). The ECM is composed of a fibrillar compartment of collagen fibrils filled with an extrafibrillar compartment, rich in proteoglycans. Unlike other connective tissues, cartilage does not contain blood vessels and is not innervated.

Therefore, oxygen and nutrient supply of chondrocytes is low and depends on diffusion from surrounding tissues, e.g., from the perichondrium. This leads to low growth rates and, after injury, to limited repair [88,89]. Therefore, and due to the lack of a sufficient number of progenitor cells, treatment of chondral defects remains a challenge to medical science. Articular cartilage lesions greater than 5 mm^2^ do not heal spontaneously. Cartilage defects are multifactorial and site-specific. Therefore, appropriate therapies need to be individualized and a clear analysis of the underlying pathology is needed. Chondral or osteochondral lesions of any type are found in 61% of patients with joint pain and are the most prevalent indications for surgical cartilage repair [90]. If left untreated, they lead, after a long asymptomatic interval, to full clinical OA.

#### 4.2.1. POMC Peptides

**Figure 2:**
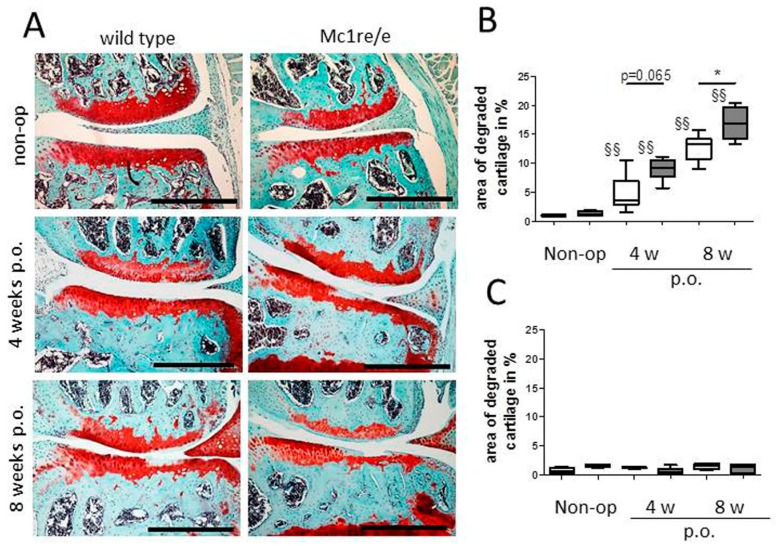
Assessment of cartilage degradation after osteoarthritis (OA) induction in Mc1re/re mice. (**A**) Representative images-stained with Safranin O/Fast green-of the medial area from frontal sections of right knee joints from non-operated (non-op) and from mice 4 and 8 weeks after osteoarthritis induction via destabilization of the medial meniscus (DMM, p.o., post-operation) show disease progression over the time. (**B**,**C**) 5–6 sections in 60–80 µm intervals of right knees (DMM, **B**) and left knees (Sham, **C**) show percentages of degraded cartilage area which indicated an OA-progression over time in wildtype (WT) (*p* = 0.0043) and Mc1re/e (*p* = 0.0095) compared to non-operated controls. Mc1re/e mice had more degraded cartilage four weeks (*p* = 0.0649) and eight weeks (*p* = 0.0411) after OA-induction compared to WT animals. Sham operated knee joints showed similar area of degraded cartilage compared to non-operated controls. White bars indicate wild type and grey bars indicate mutant group. Bars = 500 µm, §§ *p* <0.01 4/8 weeks post-surgery vs. non-operated, * *p* < 0.05 wild type vs. Mc1re/e. Adapted from Lorenz et al. [95].

Compared to what is known about POMC peptide expression and effects in synovium, much less is known regarding POMC in cartilage. Our group demonstrated the presence of MC1R, MC2R and MC5R transcripts in human articular chondrocytes derived from patients with OA [91]. Protein expression of the MC1R in these cells was confirmed in OA cartilage explants. Here, chondrocytes located in the middle and deep cartilage layers were immuno-reactive for MC1R while chondrocytes in the superficial zone were mostly negative. Treatment of these chondrocytes with α-MSH elicited a cAMP response but not a Ca^2+^ response. The detection of MC1R in human articular chondrocytes is in accordance with the observation that also a human chondrosarcoma cell line, likewise expresses functional MC1R [92]. We and others [91,93] also detected truncated forms of POMC mRNA (transcripts related to exon 2) in human articular chondrocytes obtained from patients with end-stage OA. However, POMC transcripts related to exons 2–3 were not detectable [93], suggesting that articular chondrocytes cannot make functional POMC protein. These findings suggest that expression of functional POMC transcripts in human chondrocytes is silenced by methylation of the POMC promoter [12]. In contrast to the unknown role of chondrocytes in the context of POMC peptide production, it is clearly proven that cartilage is a direct target for POMC-derived peptides. Recently, Kaneva et al. described the rapid response of articular chondrocytes to mechanical trauma—the speedy propagation of cartilage inflammation and chondrocyte death, and the ability of melanocortin peptides α-MSH and (DTRP^8^)-γ-MSH to temper this response [94]. They report that activation of both MC1 and MC3 receptor subtypes prevents the progression of trauma-induced chondrocyte death and the consequential propagation of pro-inflammatory cytokines into non-impacted areas of cartilage, concomitantly promotes the release of reparative, pro-resolving molecules. These data are in accordance with our study reporting that when challenged with OA, Mc1re/e mice (MC1R-signaling deficient mice) develop a more severe OA-pathology [95] (Figure 2). We could further demonstrate that Mc1re/e mice have a cartilage phenotype prior to OA induction that increases in severity during OA pathogenesis in an early stage. Unchallenged Mc1re/e mice display smaller articular cartilage covered areas without OA-related surface erosions, indicating that MC1R-signaling is critical for proper cartilage matrix integrity and formation. In addition, we suggest that absence of MC1R-signaling accelerates age-related structural cartilage ECM alterations as demonstrated by loss of collagen II and increased number of MMP-13 positive chondrocytes. These observations are supported by a study where adenoviral vectors encoding the human POMC gene were injected intra-articularly after surgical induction of OA by ACLT (anterior cruciate ligament transection) in rats [96]. POMC gene transfer decreased the progression and severity of OA and reduced inflammation and angiogenesis in subsynovial tissues. These effects may be triggered by inhibiting NF-κB activity and by reducing IL-1β levels like it was demonstrated in HTB-94 chondrosarcoma cells. 

#### 4.2.2. Sympathetic Neuropeptides

Although various studies verified the production of neuropeptides and neurohormones in chondrocytes, there is little evidence for NPY expression in cartilage and chondrocytes. Nunes et al. observed the expression of NPY in chondrocytes from their transthyretin (TTR) knockout (KO) mice that show overexpression of peptidylglycine α-amidating monooxygenase (PAM). NPY requires PAM for amidation to attain full biological activity and as a consequence TTR KO mice show a NPY overexpression phenotype [97]. The cartilage of these mice did not display an obviously altered phenotype. In general, research on the influence of NPY on chondrocyte differentiation exhibits considerable gaps. Furthermore, immunoreactivity against C-flanking peptide of NPY, a sympathetic marker, was detected in vascular channels of articular cartilage in OA. It is not yet clear, whether these fibers contain NPY and which role they might play [98].

Similar to NPY, little is known about PACAP-mediated effects on cartilage. PACAP-immunoreactivity was detected in cartilage canals innervating blood vessels of the femoral head and the patella from pigs [99] indicating that there might be a role for PACAP in this specific tissue. Juhasz et al. used mesenchymal cells isolated from ross hybrid chicken embryos of Hamburger–Hamilton stages to study the impact of PACAP on chondrogenesis in micromass pellets. Chondrogenic micromass cultures expressed mRNA for prepro-PACAP as well as PACAP receptor (also at the protein level) and VPAC 1 and 2 mRNA (Figure 1B). Stimulation with PACAP during culture enhanced chondrogenic differentiation and PACAP pretreatment diminished oxidative stress [100]. In a follow-up study, Juhasz and colleagues showed that mechanical load-induced hypertrophic markers were reduced by PACAP application and PACAP receptor gene expression was increased (Figure 1B) [101]. The authors did not mention any cartilage phenotype in the PACAP KO mouse [102]. Also in 2015, Giunta and colleagues published a study showing that PACAP-expressing chondrocytes and PACAP concentration in synovial fluid decreased in a rat OA model. They further showed that PACAP reversed IL-1β-induced chondrocyte apoptosis and expression of the pro-inflammatory proteins inducible NO synthase (iNOS) and Cox-2, in vitro (Figure 1B) [87]. Taken together, these few studies indicate a protective role for PACAP in chondrocyte differentiation and metabolism.

Human articular chondrocytes derived from amputation and knee replacement revealed enhanced prostaglandin E2 (PGE2) production and caseinase activity, but not cAMP production after VIP stimulation [103]. VIP concentration in synovial fluid and articular cartilage detected by ELISA and immunohistochemistry correlated negatively with OA showing high expression in controls [104]. This loss of VIP in OA-related tissue might prevent initiation of the more pro-inflammatory effects observed by Rahman et al. As mentioned above, Juhasz and colleagues observed the expression of receptors for VIP (VPAC1 and 2) in their chondrogenic micromass cultures but included no data regarding VIP-mediated effects during chondrogenesis. So far, VIP effects on chondrocytes seem controversial with upregulation of PGE2 opposed to the generally anti-inflammatory nature of this neuropeptide. Especially in collagen-induced arthritis, VIP successfully prevented cartilage and bone destruction by inhibiting the immense immune activation associated with this disease [105]. In conditions like OA, were immune activation is not the predominant impulse of disease progression, VIP and, due to the similar nature, also PACAP effects on cartilage degradation and chondrocyte behavior might be beneficial but require more intense investigations.

### 4.3. Subchondral Bone 

The subchondral bone, tightly connected to the articular cartilage, is an important shock absorber. The tissue around the interface of bone and cartilage is called the osteochondral zone. It connects the soft hyaline joint cartilage with the hard spongy bone. This connecting subchondral zone is composed of the tidemark, a thin structure between the hyaline cartilage and the subjacent calcified cartilage. Below this structure lies the subchondral bone, which blends into the spongy bone. In healthy joints, the complicated structure which is termed cartilage adjacent subchondral bone is reported to gradually relay impacting forces from the soft cartilage to the hard spongy bone [106]. Due to its intimate connection to the articular cartilage, it plays an important role in cartilage metabolism. Changes in the peri-articular and subchondral bone contribute to cartilage and joint pathology in OA. These alterations include the presence of micro-cracks, micro-edema, micro-bleeding within the subchondral region and the development of subchondral bone cysts [107]. In addition, OA progression is accompanied by the formation of osteophytes. Osteophyte formation, next to joint space narrowing, subchondral sclerosis, and subchondral cyst formation, is one of the main radiographic features and diagnostic criteria of OA [108].

#### 4.3.1. POMC Peptides 

In addition to cells of the cartilage, bone-derived cells express multiple MCR subtypes as demonstrated by independent studies. Dumont et al. reported the presence of MC4R in UMR106.06 rat osteosarcoma cells and in primary rat osteoblasts as well as in the periosteum of newborn mouse tibial bones. In primary rat osteoblasts, MC2R and MC5R transcripts were also detected (but not MC1R and MC3R mRNA) [109]. In addition, transcripts of all five MCRs were found in normal human osteoblasts as well as in MG63 and SAOS-2 osteosarcoma cells, albeit not all receptors were present in each cell type [110]. There are several reports which describe beneficial effects of POMC-derived peptides on bone structure and metabolism. ACTH reduces experimental osteonecrosis in rabbits dramatically [111]. The authors demonstrated that ACTH induces vascular endothelial growth factor (VEGF) production and supports maturation and survival of osteoblasts in vitro via the ACTH receptor MC2R. An effect on bone resorption is unlikely as osteoclasts lack MC2R expression. The same group demonstrated that ACTH regulates bone mass directly, possibly by centrally and locally produced ACTH [112]. Their work demonstrates that ACTH is capable of significantly increasing collagen synthesis in osteoblasts. They speculate that ACTH directly binds to ACTH receptors on bone cells, because direct ACTH stimulation of osteoblasts increased proliferation. Alternatively, ACTH might be acting synergistically with glucocorticoids (either locally or systemically produced) to promote osteoblastic differentiation. In addition, ACTH could also be interacting with other POMC-derived peptides (such as α-MSH or endorphins), which can bind to specific receptors and modulate osteoblastic activity and function. Contrary to ACTH, α-MSH reduces tibial perimeter and length [113]. In primary cultures of osteoblasts, α-MSH dose-dependently stimulated cell proliferation while in bone marrow cultures, α-MSH stimulated osteoclastogenesis. Systemic administration of α-MSH in mice decreased trabecular bone volume in the proximal tibiae and reduced trabecular number. From this, it can be concluded that α-MSH acts directly on bone, increasing bone turnover, and, when administered systemically, decreasing bone volume. These observations are in line with our data showing increased bone density and bone mass in MC1R-signaling deficient mice (Mc1re/e) after surgical OA-induction [95]. We observed alterations in subchondral bone micro-architecture and osteophyte numbers in Mc1re/e mice after induction of OA. Mutant mice developed clearly more and larger osteophytes compared to WT. In addition, lack of MC1R signaling led to increased subchondral bone mass and bone density after OA-induction. In line with an increase in trabecular thickness and decrease of trabecular separation, our observations indicate a more severe and clearly faster progression of subchondral OA-related sclerosis in Mc1re/e mice. Notably, we observed similar effects, however less pronounced, in sham-operated knees at four and eight weeks after surgery with increased severity in Mc1re/e mice. We were unable to detect OA-related cartilage matrix alterations in sham-knees of both groups during these time points, indicating that subchondral bone morphology alterations precede OA-related phenotypical changes in cartilage matrix.

#### 4.3.2. Sympathetic Neuropeptides

Bone cells like osteoblasts, osteoclasts and osteocytes express a wide variety of receptors for a range of neuropeptides, and substantial efforts have been made over the last decades to decipher how nervous system-derived molecules can modulate bone metabolism. VIP-positive nerve fibers were detected in the periosteum and bone of various mammalian species [114,115,116]. Additionally, several studies investigated the expression of receptors for VIP and PACAP on osteoblasts and osteoclasts from different species like human, rat and mouse (reviewed by [117,118,119]). In calvarial osteoblasts and the osteoblastic MC3T3-E1 cell line, VIP application elicited a cAMP response and induced alkaline phosphatase (ALP) gene expression and IL-6 expression [120]. Persson et al. also observed regulatory properties of VIP in osteoblast-osteoclast co-cultures and in M-CSF (macrophage colony-stimulating factor)/Rankl (receptor activator of NF-ĸB ligand)-induced osteoclastogenic cultures of bone marrow-derived macrophages (BMM). In the first, VIP delayed but enhanced osteoclastogenesis, whereas in the second setting, VIP inhibited osteoclast formation (Figure 1C) [121]. Juhasz et al. published a review on the role of VIP and PACAP in osteogenesis and chondrogenesis focusing on signaling pathways [102]. They described a complex auto-regulatory mechanism with VIP activating the transforming growth factor (TGF)-beta/ bone morphogenetic protein (BMP) signaling pathway indicating the pro-osteoblastic actions of VIP. The resulting activation of Smads then in turn regulates VIP expression. In a study from our group, we detected gene expression of VPAC receptor 1 and 2 as well as PACAP receptor 1 in mixed cultures of osteoclasts and BMMs [122]. We could not confirm that VIP had profound effects on osteoclast numbers, but we measured significantly reduced cathepsin K activity in cell culture supernatants treated with VIP. Similarly, VIP as well as PACAP38 and PACAP27, inhibited bone resorption but not number of multinuclear tartrate-resistant acid phosphatase (TRAP)-positive osteoclast-like cells in enriched cultures derived from rabbit bones [123] (Figure 1C). From the available literature, it is safe to assume that VIP acts predominantly as an anabolic factor in bone metabolism by promoting osteogenesis and reducing bone resorption. In disease conditions with increased bone resorption; VIP, therefore, has the potential to be a valuable treatment option.

Information concerning the actual distribution of PACAP in bone remains incomplete so far, except for hints that it might be released in the bone marrow and in cartilage canals near the bone [99,124]. Respective receptor expression was demonstrated in osteoblasts and osteoclasts indicating a putative role for PACAP in bone. In the rat osteosarcoma cell line UMR-106, which is capable of osteogenic differentiation, PACAP38 (agonist) and PACAP6-38 (antagonist) were able to enhance osteogenic differentiation by induction of collagen type I, osterix and ALP expression, increased nuclear expression of Runx2 and elevated deposition of inorganic matrix components into the extracellular matrix [125]. Opposite to these results, stimulation with PACAP and VIP inhibited ALP expression and stimulated bone catabolic factor IL-6 in the osteoblastic MC3T3 cell line mediated via the VPAC2 receptor [126]. Figure 1C depicts the contradictory findings of VIP-mediated effects on osteoblasts. The conflicting data might result from the different origin of cell types used in the respective studies. Probably, osteosarcoma cell lines are derived from abnormal bone cells and reactions and mechanisms studied in these cell types may, in part, not reflect physiological reactions. Apart from bone, PACAP expression was detected in osteoclasts and osteoblasts in the periodontal ligaments after tooth luxation indicating a role for this neuropeptide in the tooth microenvironment [127]. 

NPY expression was detected in the developing diaphyseal periosteum, the bone marrow, cartilage canals of epiphysis and intercondylar eminence and the bone marrow of the epiphysis emphasizing the importance of NPY during skeletal development [115]. NPY fibers associated to vessels were found in the periosteum of rat tibia as well as sporadically among bone lining cells and additionally in Volkmann canals [128,129]. Further evidence for the importance of NPY in bone and especially bone repair comes from fracture studies showing that NPY expression increased in the fracture callus and deletion of the Y1 receptor delayed healing [130,131]. NPY can not only be derived from neuronal sources but can also be produced locally by bone cells, namely osteocytes [132]. NPY promotes osteogenesis of isolated human mesenchymal stem cells by upregulation of Runx2 and ALP and the progression of differentiation is correlated with an increased expression of Y1 receptor (Figure 1C) [133] opposed to the decrease in bone mass mediated via the central Y2 receptor [134]. Studies on NPY and osteoclasts are less numerous. A study conducted by Khor et al. observed that BMM cultures from mice lacking y6 receptor expression increased M-CSF/Rankl-induced osteoclastogenesis [135]. Furthermore, Amano et al. demonstrated that NPY inhibited isoprenaline-induced osteoclast formation in mouse bone marrow cell cultures (Figure 1C) [136]. Apart from central actions, NPY might be considered as an anabolic factor in the local bone microenvironment.

## 5. Effects of the Neuroendocrine System 

### 5.1. Inflammatory and Immune Responses in OA

Clinically, OA is often accompanied by symptoms of joint inflammation, like morning stiffness, warmth, pain and joint effusions, the latter arising from synovial thickening or synovial fluid effusion [137]. OA-associated inflammation is characterized as “low-grade” thus differing from the RA-underlying “high-grade” inflammation [138]. This includes the presence of high levels of plasma proteins, complement components and cytokines in the synovial fluid and other joint tissues. Responses of the innate immune system and inflammatory mediators like the complement system, pattern recognition receptor pathways and mononuclear cell invasion are pivotal to OA inflammation. In addition to local inflammation in the joint, systemic inflammation might also play a role in OA pathogenesis. Obesity is known to predispose individuals to OA [139]—possibly not only by increasing the mechanical load on joints, but also by causing chronic, systemic inflammation through inflammatory mediators (such as adipokines and other pro-inflammatory cytokines) that are produced by adipose tissue and released into the bloodstream [140,141]. Weight loss is associated with a substantial reduction in systemic levels of C-reactive protein and IL-6 in individuals with OA, and can prevent OA onset or alleviate existing OA symptoms [142,143]. It is possible that the systemic inflammation associated with chronic inflammatory states, such as obesity or certain chronic diseases, promotes local inflammation in joints that ultimately results in OA [138]. However, this offers the opportunity to develop DMOADs for OA that target inflammatory mediators and pathways.

#### 5.1.1. POMC Peptides 

A key mediator of cartilage degradation is MMP-13 with clearly elevated levels in joint disorders. Yoon et al. demonstrated that 200 nM α-MSH inhibits tumor necrosis factor (TNF)-induced MMP-13 expression by decreasing p38 kinase phosphorylation and, thus, preventing subsequent activation of the NF-kB pathway in HTB-94 chondrosarcoma cells [92]. This is in line with an observation of our group that α-MSH reduced secretion of pro-MMP-13 and pro-MMP-2 in addition to suppressing mRNA levels of IL-1β and TNF in articular OA chondrocytes [91]. Besides, we demonstrated that α-MSH stimulation of primary mixed synoviocytes and OASFs reduced TNF, IL-6 and IL-8 secretion [64]. Furthermore, MC1R is the only receptor for α-MSH and related peptides in OASFs. That way the receptor defines a viable target by which anti-inflammatory therapies could be delivered to potentially treat inflammatory and degenerative joint diseases.

Transgenic mice overexpressing N-terminal POMC, including α-MSH and γ_3_-MSH, were crossed with obese leptin-receptor-deficient mice [144]. Interestingly, the results showed that MSH overexpression was effective in reducing weight gain and adiposity plus improving glucose tolerance and insulin sensitivity in lean mice and in genetic mouse obesity models. These results provide support for the hypothesis that long-term melanocortin activation and treatment could serve as a potential strategy for anti-obesity therapy subsequently also ameliorating OA pathology and progression. 

Intra-articular injection of the POMC gene by using an adenoviral vector carrying the human POMC gene, inhibited inflammation and angiogenesis in ACLT-induced OA in rats [96]. In addition to anti-inflammation, other mechanisms may participate in OA suppression by the POMC gene transfer. Osteochondral angiogenesis causes cartilage loss, osteophyte formation and synovial inflammation, and facilitates the progression of OA [145]. POMC gene transfer may regulate the expression of the angiogenic factor VEGF by inhibiting NF-kB activity. Other evidence indicates that POMC gene delivery inhibited the migration and tube formation capability of endothelial cells [146]. The derived neuropeptides ACTH and β-EP are also anti-angiogenic [147]. 

Recently, the group of Perretti performed compound screening in order to identify novel agonists or positive allosteric modulators (PAM) of the human MC3R [148]. They identified fenoprofen (a potent cox inhibitor) as a ligand of MCRs (MC3, MC4 and MC5). The novel aspect of these findings is that a drug with indication for RA and OA might be engaging the melanocortin system, providing proof-of-concept for MC3R targeting as a treatment for joint diseases. They conclude that targeting MC3R using PAMs constitutes a viable and biologically effective means to reduce synovial inflammation.

An interesting approach to target inflammatory arthritic diseases was published by Vessellier et al. The group generated latent forms of VIP, α-MSH and γ3-MSH by fusion to latency-associated peptide (LAP from TGF-β) through an MMP cleavage site using recombinant DNA technology [149]. A major limitation for usage of melanocortins as anti-inflammatory drugs in the clinic is their short half-lives. The authors demonstrated that these anti-inflammatory peptides can be made latent by insertion into the LAP shell of TGFβ protecting them from degradation until they reach the site of inflammation where they can be released by MMPs. In the same line, anti-arthritic effects of a more stable MSH-based compound, (DTrp^8^)-γMSH, were associated with protection against alveolar bone loss that is typical for arthritic mice [150]. Furthermore, in line with data obtained from bone-marrow-derived osteoclasts, a physiologic control of osteoclast reactivity was observed exerted by MC3R by demonstrating that Mc3r-null mice develop periodontal disease with accelerated kinetics as they age [151]. Recently, the group demonstrated that periodontal tissues infected with *A. actinomycetemcomitans* increased expression of MC3R, which upon activation reduced alveolar bone loss [152]. Treatment with (DTrp^8^)-αMSH reduced the number of TRAP-positive osteoclasts and decreased production of TNFα, interferon (INF)-γ and IL-17A. In addition, (DTrp^8^)-αMSH impaired bone resorption by reducing the number of resorptive pits.

#### 5.1.2. Sympathetic Neuropeptides

VIP and PACAP play important roles in the immune system. Numerous cells of the immune system are able to produce and secrete VIP, and are modulated by the neuropeptides themselves through expression of the respective receptors. The presence of VIP-positive nerves in the thymus, spleen, lymph nodes and mucosal-associated lymphoid tissue further supports the important role of VIP in immunity [153,154]. PACAP has been detected in cells of lymphoid organs like bone marrow, spleen, lymph nodes and gut mucosa of the rat by immunostaining, and also in isolated murine lymphocytes [155,156]. A very comprehensive set of reviews addressing the effects of VIP and PACAP on immune cells, comes from Mario Delgado and Doina Ganea. Both researchers emphasize the strong anti-inflammatory nature of both peptides on cells of the innate and adaptive immune system although PACAP-related effects require more context-dependent interpretation [157,158,159]. More recently, it was reported that VIP prevented acquisition of the pro-inflammatory phenotype in macrophages [160] and inhibited reactive oxygen species and extracellular trap formation in neutrophils from healthy volunteers [161]. VPAC1 is the predominant receptor mediating immune regulatory functions although studies from VPAC2 and PAC1-deficient mice show increased susceptibility to inflammatory disorders. In humans, reduced levels of VPAC1 on immune cells as well as a reduced response to VIP stimulation were reported in ankylosing spondylitis, multiple sclerosis, RA and OA [81,162,163,164]. VIP-based therapies were especially effective in collagen-induced arthritis [105,165] and its therapeutic potential has been evaluated in a large number of experimental pathological conditions including inflammatory bowel disease [166], allergic asthma [167], wound healing [168] and stroke [169]. Scarce availability of the PACAP receptor 1 might indicate that the role of PACAP is less prominent or more specialized compared to the structurally related VIP. Similar to VIP, PACAP was able to ameliorate disease outcome in a murine collagen-induced arthritis model and was very effective in preventing septic shock after several lipopolysaccharide (LPS) injections in a murine model [159]. Observations from Liu et al. show that anti-inflammatory M2 macrophages were able to induce PACAP expression in neural stem/progenitor cells adding to the anti-inflammatory microenvironment [170].

Like VPAC and PACAP receptors, NPY receptors, especially the Y1 subtype have been detected on almost every type of immune cell so far [171,172]. The aforementioned review highlighted the role of NPY in numerous inflammatory conditions like inflammatory bowel syndrome, ulcerative colitis, liver cirrhosis, asthma and atopic dermatitis, as well as autoimmune conditions including diabetes type I, systemic lupus erythematosus, rheumatoid arthritis and multiple sclerosis. NPY seems to play an important role in the regulation of phagocytosis and resolution of pathogen invasion mediated by macrophages and neutrophils [173,174]. Anti- as well as pro-inflammatory effects of NPY were described in macrophages and dendritic cells indicating the context-dependent role of NPY in immunity [35,175]. Rather than being a constitutive agent in immune function, NPY might be an inducible modulator in various immune cells [176,177]. NPY seems to play an important role during inflammation in fracture healing where it increased in the serum of fractured rats. Additionally, fracture healing was inhibited by administration of specific receptor antagonists. Immunohistochemistry revealed accumulation of NPY- and calcitonin gene-related peptide (CGRP)-positive macrophages in the callus region possibly initiating fracture healing by neuropeptide-induced activation of the ERK signaling pathway [178]. NPY actions might also include preservation of a stable microenvironment in the bone marrow niche thereby securing the pool of immune cell progenitors [179,180]. The stem cell regulatory functions of NPY could highlight the potential of this peptide as a modulatory supplement in stem-cell-based therapeutic regimens for a range of diseases e.g., osteoporosis (reviewed by Peng et al. [181]). Regarding OA, studies of the impact of NPY on the immunologic and degradative mechanisms are lacking and predominantly indicate that NPY participates in pain mechanisms [76]. These diverse findings suggest highly specific NPY immune actions including pro- and anti-inflammatory mechanisms that might target very distinctive features of the immune response. Evaluation of NPY effects might also need to be evaluated in the context of higher hierarchies of the immune response rather than just the cellular level.

## 6. Neuroendocrine Peptides: Tools for Treatment of OA? 

Current treatments for OA are not regenerative and have little impact on the progressive degeneration of joint tissues. Clinical interventions are primarily symptomatic with a focus on pain reduction and control of inflammation with non-steroidal anti-inflammatory drugs and ultimately through total joint replacement [182,183]. One major reason for this under-representation of regenerative therapies is, as opposed to isolated focal articular cartilage defects, that regenerative strategies have to take into consideration the larger sizes of the defects, as well as the underlying disease process [184]. Fragile neocartilage constructs produced by implanted or injected mesenchymal stem cells (MSC) or chondrocytes, together with anti-inflammatory/tissue-protective molecules, may face rapid degradation when situated in inflamed or diseased joints. Therefore, the underlying pathology has to be brought effectively under control or any regenerative treatment strategy of OA is rather short-term and unlikely to be successful long-term. In addition, patients with OA represent a heterogeneous population in terms of the underlying pathophysiology [185]. This knowledge implies that joint repair lacks a one-for-all therapy. OA can be divided based on pathophysiological phenotypes (i.e., bone-, cartilage-, or inflammation-focused) which may affect progression rates. Biomarkers are needed for the identification of fast-progressors and patient phenotypes so that appropriate patient populations can be selected for clinical trials and tailored treatments. 

### 6.1. POMC Peptides

**Figure 3:**
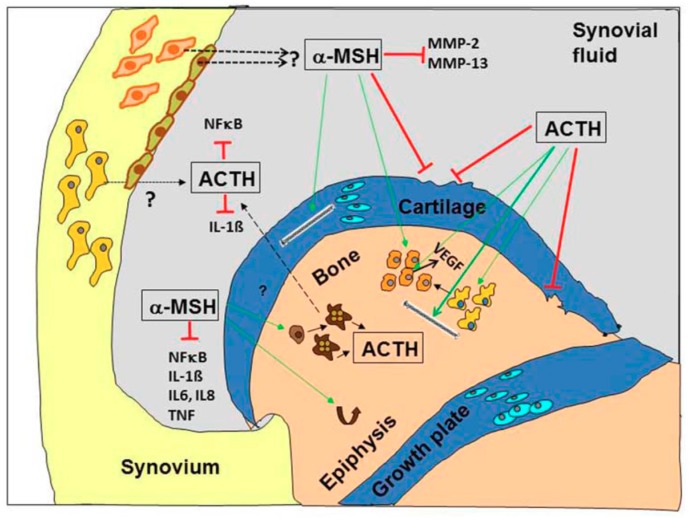
Molecular targets and anti-inflammatory mechanisms of melanocortin peptides in the joint. Proopiomelanocortin (POMC)-derived peptides affect several cellular and molecular targets in the synovium, bone and articular and growth cartilage in the joint. Adrenocorticotropin (ACTH) induces bone matrix production by increasing collagen expression, it induces proliferation of chondroprogenitor cells, it promotes chondrocyte differentiation from progenitor cells, it modulates osteogenic differentiation, and it protects against osteonecrosis by stimulating vascular endothelial growth factor (VEGF) production and it inhibits nuclear factor “kappa light chain enhancer” of activated B cells (NF-κB) pathways and interleukin (IL)-1β expression. α—melanocyte-stimulating hormone (MSH) induces cartilage matrix production (collagen II and aggrecan), it increases proliferation of osteoblasts and stimulates osteoclastic differentiation from precursors, it induces bone turnover of trabecular bone and it inhibits cytokine-induced matrix metalloproteinase (MMP)-2 and MMP-13 expression and IL-1β, IL-6, IL-8, TNF expression and blocks NF-kB pathways. Black arrows indicate origin of POMC peptides within the joint. α-MSH is secreted presumably from CD68-positive synovial cells and endothelial cells and ACTH from CD68-negative synovial fibroblast and presumably from osteoclasts (dotted black lines with question mark). Green arrows indicate stimulatory effects culminating in cell differentiation, proliferation and modulation of gene expression; red bars indicate inhibition and the brown curved arrow indicates bone turnover. Adapted from Böhm and Grässel [12].

The important question arises if POMC-derived peptides and derivatives are promising future candidates for the treatment of OA. As described above, melanocortin peptides target osteoarticular key cells in inflammatory diseases—including OA- and modulate pathogenetically relevant key players including pro-inflammatory cytokines, MMPs, NF-κB or Rankl (Figure 3).

Melanocortins could have several advantages over currently used anti-inflammatory drugs as they can modulate, but not abrogate, the response of a broad number of cell types. Clinically, administration of melanocortin peptides to patients with inflammatory (or degenerative) joint disease may be a priori more advantageous than application of opioids due to the addictive character of the former agents. The fact that melanocortins not only target virtually all cells of the osteoarticular system but also those of the immune system [16,186], makes these peptides even more attractive as future therapeutic candidates for OA. Melanocortin can regulate the inflammatory response without the risk of side-effects observed with immunosuppressive therapies. Importantly, melanocortin peptides—albeit targeting immune cells—are rather immunododulators than immunosuppressants. Notably, the melanocortin analog (DTrp^8^)-γMSH reduced alveolar bone loss after infection with a Gram-negative coccobacillus by preventing overexuberant inflammatory responses and bone resorption via osteoclasts [152]. These data highlight the potential of POMC-derived peptides to target the inflammatory processes and bone-related processes in OA pathology. Furthermore, MSH peptides might be especially useful in obesity-induced OA. Overexpression of N-terminal POMC in obese leptin receptor-deficient mice prevented obesity and preserved normal energy metabolism [144]. Obesity is additionally associated with a higher individual inflammatory status [187] and thus POMC-derived peptides might be especially beneficial in obese OA patients due to their ability to modulate metabolic and anti-inflammatory properties. However, one major challenge encountered with the clinical use of melanocortin peptides is their rapid degradation in vivo [12]. Oral administration of natural melanocortin peptides therefore seems to be impossible. Even intravenously administered full-length α-MSH has a half-life of only a few minutes [188] due to the action of serum proteases [189]. Therefore, development of protease-stable MSH analogues with MCR selectivity, increased potency but maintained anti-inflammatory efficacy, is a prerequisite to bring melanocortins successfully into the clinic. In that line, the group of Perretti has undertaken a successful approach to ameliorate experimental arthritis in mice [149]. They generated a fusion protein consisting of LAP from TGF-β and γ_3_-MSH which exerted anti-inflammatory activity and had a half-life of more than 30 h. Finally, a more promising but challenging strategy in the treatment of inflammatory (or degenerative) joint diseases may be the exploitation of protease-resistant small peptide derivatives from the C-terminal domain of α-MSH. MSH tripeptide derivatives such as KdPT do not bind to the MC1R but have potent anti-inflammatory effects against IL-1β in sebocytes [190]. 

In this context it will be necessary to clarify which specific MCRs or ORs confer a chondroprotective function in experimental OA models and in human OA. We have shown that in an MC1R signaling deficient mouse strain, OA progression was faster and OA scores were more severe compared to the wildtype (WT) controls indicating a pivotal role of the MC1R in cartilage and subchondral bone homeostasis [95]. In addition, the MC3R signaling is implicated in tissue protection and thus MC1R and MC3R targeting might also qualify as treatments of joint diseases [148].

In conclusion, the use of POMC-derived peptides in OA is most promising when targeting the ongoing inflammatory processes. The peptides seemingly preserve cartilage and bone structure, but the exact mechanisms remain uncertain at this point. 

### 6.2. Sympathetic Neuropeptides

From all the evidence presented above, NPY, VIP and PACAP can surely be considered to have therapeutic potential in OA. OA is a multi-factorial disease with diffuse and complex etiology. Neither of the neuropeptides may be adequate to completely cure or prevent OA, but certain aspects could be targeted and improved such as pain, cartilage degradation, osteophyte formation or bone alterations. The greatest potential for a beneficial therapeutic effect in OA might be attributed to VIP. In fact, Jiang et al. reviewed the therapeutic potential of VIP in OA and concluded that VIP offers an interesting treatment option, but some drawbacks necessitate more intense studies first [191]. In a study of murine collagen-induced arthritis, VIP was able to abrogate inflammation as well as bone and cartilage destruction [105], thus identifying VIP as a perfect candidate for application in OA. Although OA and RA share certain features like the destruction of cartilage and alterations of bone, RA remains a disease that is massively driven by autoimmune reactions that might be dissolved by VIP administration [157]. The immune system participates in OA disease pathology but is not the main driving force of structural deterioration. Bone destruction in RA is also a result of the massive expression of the osteoclastogenic factor receptor Rankl [192] which can also be down-regulated by VIP [193]. Accumulating evidence indicates a role for abnormal bone homeostasis in OA due to a dysregulated Rankl/osteoprotegerin balance [194] that could be addressed by VIP-based drugs. Lower VIP synovial fluid concentrations negatively correlated with the Kellgren–Lawrence score and VIP cartilage content negatively correlated with the histological Mankin score in OA [104]. Thereby, loss of VIP might contribute to OA pathogenesis and restoration of VIP levels might potentially halt or abrogate disease progression. Supporting this thesis are several studies demonstrating that VIP has beneficial effects on synovial cell-derived joint morbidities. Synovitis is a frequent feature of early and late stage OA that involves infiltration of mononuclear cells, production of pro-inflammatory cytokines like IL-1β, IL-6 and TNF as well as expression of various matrix-degrading MMPs in the synovial fluid of OA patients [137,195]. Synovitis and the coherent expression of deleterious molecules might be prevented by VIP administration [80]. VIP levels of RA synovial fibroblast-like cells (RASF) were reduced compared to OASF, and VIP stimulation was able to inhibit the highly aggressive destructive phenotype of RASF [81,82,83,84,196]. Reduction of inflammatory molecules and degradative enzymes would also benefit cartilage preservation. VIP is able to target inflammation and bone metabolism and its application might thus be suitable for specific OA subtypes. One drawback of peptidergic therapy lies in the short half-life of the peptides that rapidly reduces bioavailability. Possible solutions include the packing of peptides into self-assembling micelles thus prolonging their lifespan and delivery of the micelles directly into the joint cavity. A successful therapeutic approach with VIP packed into self-assembling micelles was established in a collagen-induced arthritis model and abrogated joint swelling, cartilage and bone destruction [165]. A major obstacle for VIP as a therapeutic agent in OA is the possibility that it might promote knee joint allodynia and secondary hyperalgesia in OA joints, an effect that might also be provoked by administration of PACAP [197,198]. The potent vasodilatory properties of VIP might also promote unwanted side effects by enhancing immune cell influx into the joint region. 

Similar to VIP, PACAP was described to ameliorate inflammation and cartilage and bone destruction in a collagen-induced arthritis model probably due to its modulatory role in immune-related reactions [159,199]. The direct chondroprotective effects of PACAP make it even more considerable as a therapeutic for OA [87]. More recently, the group of Botz et al. used the serum-transfer arthritis model in PACAP-deficient and WT mice to elucidate the role of PACAP in inflammatory arthritis and found some surprising stage-dependent effects. In early arthritis, PACAP-deficiency ameliorated, and in a later stage it aggravated, arthritis in this model [200]. Hence, PACAP might not only have beneficial effects, and intention of therapeutic use requires thorough studies of PACAP availability and specific receptor expression during every stage of OA. More intense studies should aim to further elucidate the role of the PAC1 receptor. If the VPAC receptors also transduce signals leading to deleterious effects like afferent nerve sensitization, activation of the PAC receptor while inhibiting the others might be an option to transmit the beneficial effects of PACAP.

Likewise, NPY synovial fluid concentrations correlated with Watanabe pain score in OA patients and therefore NPY needs further critical evaluation before being considered as a therapeutic option or should be preferably considered as a biomarker candidate in OA. 

Irrespective of the potential structure-preserving properties of VIP and PACAP, local sensitization of afferent nerves needs to be prevented before considering them as drugs. In order to prevent hyperalgesia, it might be necessary to inhibit certain receptors while activating others leading to the development of combinational therapies. Much more work lies ahead in the future before sympathetic neuropeptides can be considered in OA therapy.

## 7. Conclusions

OA is a heterogeneous disease of all joint tissues and not only of the articular cartilage. Based on the pathology, OA can be divided into at least three phenotypes which are bone-, cartilage- or inflammation based that may affect progression and severity of the disease. Besides, future treatment strategies have to take into account other problems of novel drugs like target-specificity and side-effects. Neuropeptides such as VIP and NPY and neuroendocrine peptides such as proopiomelanocortin (POMC)-derived peptides are promising candidates for future treatment options of OA. POMC-peptides, specifically melanocortins, are partly osteo- and chondro-protective and all of them show immunomodulatory effects dampening inflammatory processes (Figure 3). Thus, local application of POMC-derived peptides, i.e., α-MSH, may serve as a potential adjuvant therapy for delaying the process of posttraumatic OA. However, before the clinical application of these compounds, problems associated with half-life and stability as well as application strategy (oral versus intra-articular) have to be solved. Research on the protective role of VIP on different joint tissues is predominantly restricted to studies in inflammatory arthritis models. Putative effects in OA are of great interest and deserve more intense investigations because available studies so far emphasize VIP as a potential inhibitor of joint degradation. From the available literature, it is safe to assume that VIP acts predominantly as an anabolic factor in bone metabolism by promoting osteogenesis and rather reducing bone resorption. Therefore, in disease conditions with increased bone resorption VIP has the potential to be a valuable treatment option. 

In conclusion, neuropeptides and neurohormones are a promising source for novel DMOADs but major obstacles like their quick turnover time and costly production need to be addressed before they can be considered comparably effective like small molecules including glucosamine derivatives.
